# Human Gut Microbiota: Repertoire and Variations

**DOI:** 10.3389/fcimb.2012.00136

**Published:** 2012-11-02

**Authors:** Jean-Christophe Lagier, Matthieu Million, Perrine Hugon, Fabrice Armougom, Didier Raoult

**Affiliations:** ^1^URMITE, UM63, CNRS 7278, L’Institut de Recherche pour le Développement 198, INSERM 1095, Aix-Marseille UniversitéMarseille, France

**Keywords:** gut microbiota, culturomics, metagenomics, archaea, transparency disclosures, antibiotics

## Abstract

The composition of human gut microbiota and their relationship with the host and, consequently, with human health and disease, presents several challenges to microbiologists. Originally dominated by culture-dependent methods for exploring this ecosystem, the advent of molecular tools has revolutionized our ability to investigate these relationships. However, many biases that have led to contradictory results have been identified. Microbial culturomics, a recent concept based on a use of several culture conditions with identification by MALDI-TOF followed by the genome sequencing of the new species cultured had allowed a complementarity with metagenomics. Culturomics allowed to isolate 31 new bacterial species, the largest human virus, the largest bacteria, and the largest Archaea from human. Moreover, some members of this ecosystem, such as Eukaryotes, giant viruses, Archaea, and Planctomycetes, have been neglected by the majority of studies. In addition, numerous factors, such as age, geographic provenance, dietary habits, antibiotics, or probiotics, can influence the composition of the microbiota. Finally, in addition to the countless biases associated with the study techniques, a considerable limitation to the interpretation of studies of human gut microbiota is associated with funding sources and transparency disclosures. In the future, studies independent of food industry funding and using complementary methods from a broad range of both culture-based and molecular tools will increase our knowledge of the repertoire of this complex ecosystem and host-microbiota mutualism.

## Introduction

**Figure 1:**
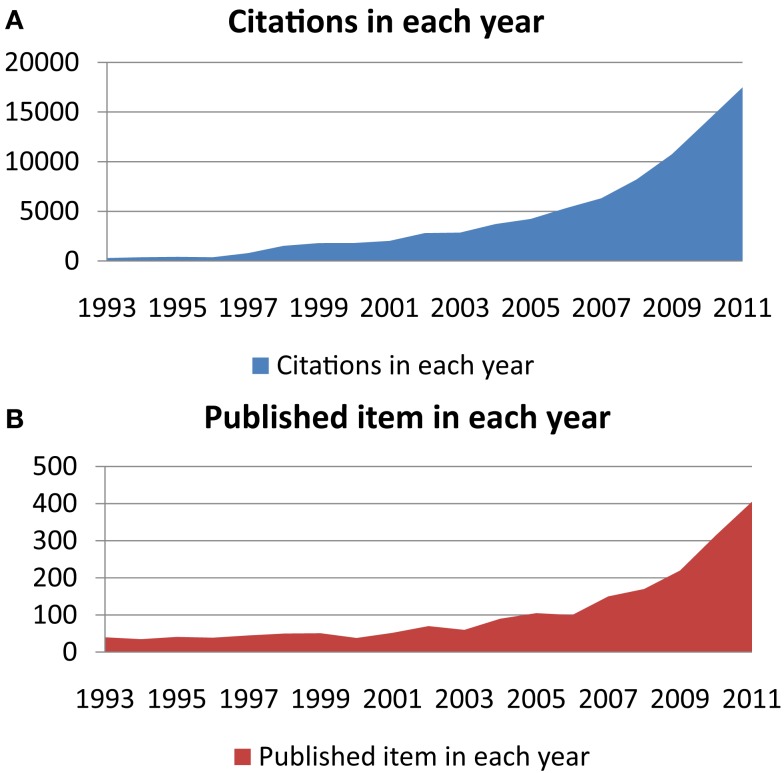
**Using the key words “human gut microbiota” or “human fecal flora” and using the ISI Web of Knowledge database, (A) shows citations in each year regarding this subject, and (B) shows the number of published items each year, both between 1993 and 2011**.

The exhaustive description of human microbiota and their relationship with health and disease are major challenges in the twenty-first century (Turnbaugh et al., 2007). To assess the importance of this challenge, we used the ISI Web of Knowledge to demonstrate the dramatically renewed interest of scientists in this subject. To extend the chart presented by Sekirov et al. (2010); Marchesi (2011), which lists the number of publications per year involving human gut microbiota, we found that in 2011, there were more than 4 times as many citations referencing human gut microbiota than in 2005 (Figure 1A), when Eckburg et al. (2005) published the seminal large-scale gut metagenomics study. In addition, in 2011, there were approximately as many published items investigating human gut microbiota than during the 10 years between 1993 and 2002 (Figure 1B).

The human gut microbiota is composed of approximately 10^11–12^ microorganisms per gram of content, including diverse populations of bacteria, mainly anaerobes (95% of the total), which is 10 times higher than the total number of human cells (Ley et al., 2006a). In the study of human gut microbiota, two major technological periods can be distinguished: schematic microscopic observation and culture-based methods before 1995 followed by the advent of culture-independent methods. This technology-driven progress led to suggest relationships between gut microbiota composition and diverse diseases, such as irritable bowel syndrome (Kassinen et al., 2007), polyposis or colorectal cancer (Scanlan et al., 2008), necrotizing enterocolitis (Siggers et al., 2008), Crohn’s disease (De Hertogh et al., 2006; Manichanh et al., 2006; Scanlan et al., 2006), and metabolic diseases such as type II diabetes (Larsen et al., 2010) and obesity (Ley et al., 2006b; Turnbaugh et al., 2006, 2009; Armougom et al., 2009; Santacruz et al., 2009).

Based on these early data and to complete the description of the human gut composition, considerable funds have been granted. Among the projects pursuing this line of research, the human microbiome project is an international consortium with the aim of sequencing 1,000 bacterial genomes and multiplication by metagenomic analysis to characterize the complexity of microbial communities at several body sites, including the human gut, to determine whether there is a core microbiome (Turnbaugh et al., 2007). Despite these advances in knowledge of gut microbiota composition, the relationships of the microbiota with their host and, consequently, with health and disease are still largely unknown, as reflected in several contradictory results (Sekirov et al., 2010). Moreover, molecular tools and by extension, experimental models, often reflect a reductionist approach as opposed to a holistic approach (Fang and Casadevall, 2011). Nevertheless, an appealing approach that was recently applied to the study of oral microbiota will allow us to detect the minor bacterial populations, which are usually neglected, using dilution to obtain a threshold below 10^6^ bacteria per ml or DNA >1 pg per μl (Biesbroek et al., 2012).

We propose here an inventory of current knowledge regarding gut microbiota composition, the techniques used for this study and the relationships with the host. Finally, further research on human gut microbiota is the subject of considerable funding by the food industry. Consequently, to perform an efficient analysis of this subject, the design and/or interpretation of the results of each study can be associated with a conflict of interest. For example, it has recently been shown that published papers in obesity research in which the authors were funded by the food industry were more likely than other papers to contain results or an interpretation that favored the industry or company that was producing the product or service that was being studied (Thomas et al., 2008).

## Repertoire

### Culture

Culturing has been the first method used to characterize a bacterial ecosystem (Finegold et al., 1974, 1977; Moore and Holdeman, 1974a). Gut composition was first studied by microscopic observation and axenic culture. Gram staining has been widely used by microbiologists to describe stool composition. Using this technique, gram-positive bacteria accounted for only 2–45% of the cells observed (Gossling and Slack, 1974). However, a discrepancy arises because culture counts reveal a predominance of gram-positive bacteria in human feces. Indeed, one of the first culture studies of human stools showed that anaerobes always constitute the major component of the culturable flora of children and adults (Mata et al., 1969), with a predominance of gram-positive cells. Moore and Holdeman (1974a), in a study of 20 individuals, revealed 113 different bacteria, including more gram-positive bacteria (*Bifidobacterium*, *Eubacterium*, *Peptostreptococcus*, *Ruminococcus*, *Lactobacillus*, and *Clostridium* genera) than gram-negative bacteria (*Bacteroides*, *Fusobacteria* genera;). Nevertheless, these studies attempted especially to culture anaerobic bacterial species whereas some gut bacteria preferentially grown in microaerophilic conditions.

**Figure 2:**
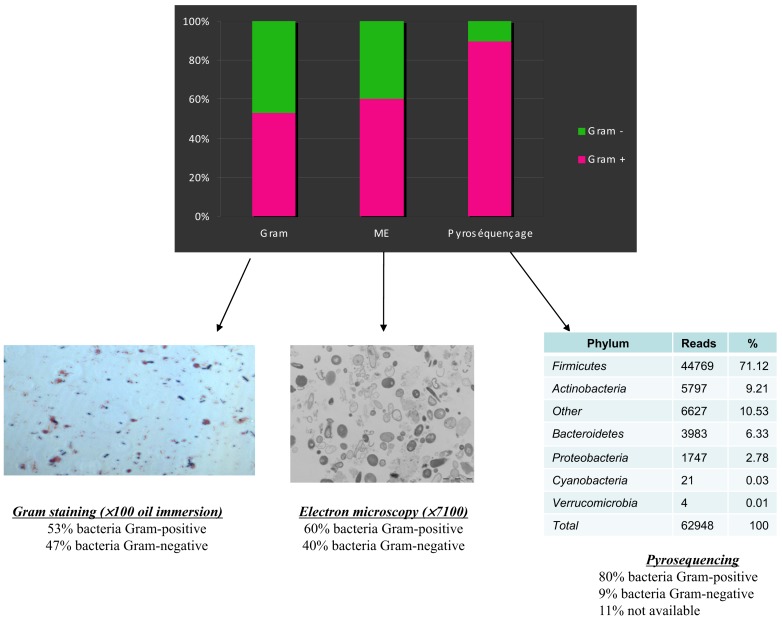
**A comparison of Gram staining, electron microscopy, and pyrosequencing to determine the proportion of Gram-positive/Gram-negative bacteria in the same stool sample (personal data)**.

Among other unique problems associated with bacterial culture, Moore have also observed a major discordance between the culture counts and the microscopic counts of species (Moore and Holdeman, 1974b); these discrepancies have been named by Staley and Konopka (1985) as the “great plate count anomaly”. Indeed, it is generally accepted that only 1% of bacteria can be easily grown *in vitro* (Vartoukian et al., 2010). Consequently, the major population easily isolated from stools is composed of bacteria that grow quickly in classical high-nutrient growth media, with the usual carbon or electron sources at mesophilic temperatures (Hugenholtz, 2002), and this constitutes the most studied bacteria. It is estimated that approximately 75% of published studies by microbiologists before the advent of molecular tools focused on only nine bacterial genera among four phyla (*Actinobacteria*, *Proteobacteria*, *Firmicutes*, *Bacteroidetes*; Galvez et al., 1998), whereas we know now that more than 30 different phyla compose the gut microbiota (Figure 2; Rajilic-Stojanovic et al., 2007). Nevertheless, studies of these fast-growing and easily cultured bacteria neglect the minority bacterial populations, including potentially pathogenic bacteria such as *Salmonella typhi*.

Finally, considering the main first culture-based studies the number of bacterial species was estimated at approximately 400–500 (Mata et al., 1969; Moore and Holdeman, 1974a; Finegold et al., 1977). In addition to the necessary use of stringent anaerobic conditions to culture bacteria from human stools, the usual phenotypic identification methods are time consuming and expensive (Seng et al., 2009). Indeed, the exponential technological advances in molecular tools led microbiologists to progressively abandon the culture-based approach for studies of the gut microbiota ecosystem.

### Metagenomics and pyrosequencing

As often occurs during scientific progress, technological advances in microbiology allowed scientists to revisit the knowledge base (Rajilic-Stojanovic et al., 2007). Since 2000, large-scale 16S rRNA or metagenomic studies have allowed scientists to dramatically expand the known diversity of the human gut microbiome, illuminating new ways (Eckburg et al., 2005; Andersson et al., 2008). It is now commonly accepted that approximately 80% of the bacteria found by molecular tools in the human gut are uncultured, and hence can be characterized only by metagenomic studies (Eckburg et al., 2005). Whereas the number of species was limited in the seminal studies using culture-based methods (Finegold et al., 1974; Moore and Holdeman, 1974b), Turnbaugh et al. (2010) estimated 473 phylotypes using V2 pyrosequencing. There is a significant discrepancy between bacterial observations with a microscope and most of the molecular studies, which observe a striking dominance of gram-positive bacteria (Eckburg et al., 2005; Andersson et al., 2008; Turnbaugh et al., 2010; Figure 2).

Indeed, these recent methods generate contradictory results reflecting the biases in every step of the Polymerase Chain Reaction procedure. A dramatic divergence in the proportion of the different phyla was observed depending of the type of extraction kit used, notably for the *Fusobacteria* (2–40%) and *Bacteroidetes* (40–60%) phyla (Wu et al., 2010). In addition, the relative abundance of a phylum depends significantly on the 16S hypervariable region, independent of pyrosequencing chemistry. For example, the 454 titanium and Illumina next-generation sequencing (NGS) methods reveal a dominance of the *Bacteroidetes* phylum using 16S rDNA v4v5 region primers, whereas *Firmicutes* was predominant using v3v4 primers on the same gut microbiota (Claesson et al., 2010). Using 454 titanium, *Ralstonia* genera have been detected only by V4/V5 primers, whereas *Bifidobacteria* have been detected only by V3/V4 primers (Turnbaugh et al., 2010). In parallel, Hong et al. (2009) have described that the rRNA approach misses half of the bacteria in environmental microbiology.

Although controversial, the higher taxonomic level analyses (as phylum level) have suggested an association between obesity and *Firmicutes/Bacteroidetes* proportion (Ley et al., 2005). The genus-level analysis has allowed to hypothesize specific enterotypes compositions despite controversies (Arumugam et al., 2011). In addition, Murphy et al. (2012) has recently observed in a study from the manipulation of the mice gut microbiota in diet-induced obesity that a better separation of lean and diet-induced obese mice was observed at the family and genus-level than at the phylum level. However, the large inter-individual variability leads the analysis of lower taxonomic-level to complex results because of small number of samples. Finally, the optimization of primers able to detect genera often misdetected by pyrosequencing, as *Bifidobacteria* (Sim et al., 2012), and technology progress in pyrosequencing, will allow to more quickly analyze longer reads sequenced to study larger cohort samples in low taxonomic level.

**Figure 3:**
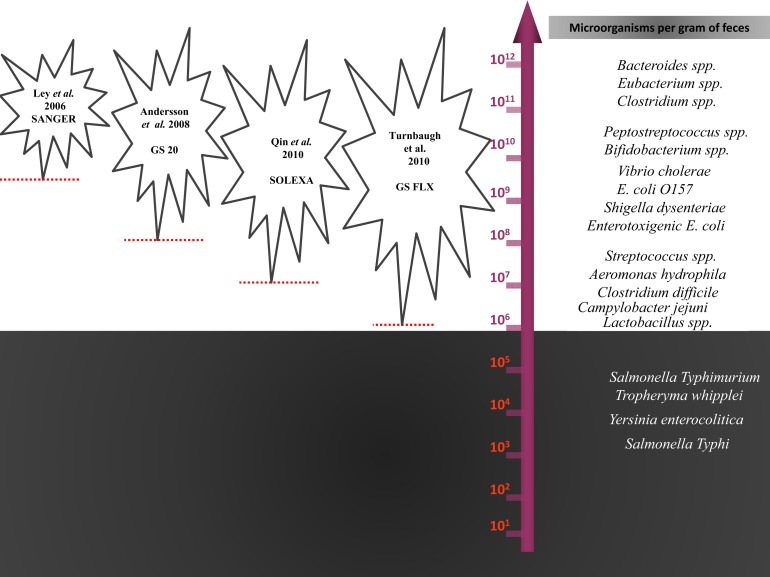
**The statistical detection thresholds of metagenomic methods**. The statistical detection thresholds of metagenomic methods are correlated with the number of bacteria in the ecosystem studied by the number of sequences generated.

Finally, molecular methods detected bacteria present at concentrations greater than approximately 10^6^ and neglected minority populations. Among these neglected populations are potentially pathogenic bacteria such as *S. typhi*, *Yersinia enterocolitica*, and *Tropheryma whipplei*, which may be present in human stools at concentrations below 10^5^ cfu per ml (Raoult et al., 2010), the current threshold of the latest NGS method (Turnbaugh et al., 2010; Lagier et al., 2012a; Figure 3). The depth is directly correlated with the number of generated sequences, and no plateau was obtained in the number of phylotypes observed, although close to 1,000,000 16S rRNA gene amplicons have been sequenced by Turnbaugh et al. (2010).

### Viruses

Research in the human gut has been focused on bacterial composition (Walker, 2010). Early studies suggested that most DNA viruses found in the intestine were phages and that most RNA viruses were plant viruses (Breitbart et al., 2008). Nevertheless, a recent metagenomic study carried out over 1 year, with three stools analyzed from each monozygotic adult twin and their mother, revolutionized virome knowledge (Reyes et al., 2010). The authors carried out shotgun pyrosequencing to generate over 280 Mb of sequence and, at the same time, a pyrosequencing of 16S rRNA genes to identify the bacterial species. Approximately 80% of sequencing reads did not match any known viruses in the database corresponding to prophages or temperate phages. These populations were persistent in each individual, with no significant clustering between co-twins or between twins and their mothers, contrasting with the bacterial similarity between twins (Turnbaugh et al., 2009). In addition, Minot et al. (2011) observed that a change of diet is associated with a change in virome composition.

### Culturomics

There has been a renewed interest in culture methods for these “non-cultivable” species (Vartoukian et al., 2010). Initially, environmental microbiologists were confronted with the fact that the majority of bacteria do not grow in classical Petri dishes. These first studies used prolonged incubation and stringent anaerobic conditions, notably, diffusion chambers (Kaeberlein et al., 2002; Bollmann et al., 2007), with the aim of simulating the natural environment of these “uncultivable” microorganisms (Kaeberlein et al., 2002). This technique enlarged the diversity of the environmental microorganisms that were isolated (Epstein, 2009). In parallel, a recently published study proposed an anaerobic culture of a single stool sample to complement 16S rRNA sequencing, using rumen fluid or an extract of fresh stools to mimic the natural environment of the gut bacteria. Goodman et al. (2011) have recovered 36 cultured species: four uncultured described species and 53 unknown isolates with different v2 sequences. However, these authors used the most probable number (MPC) technique for creating arrayed species collections that do not detect minority populations.

In addition to the stringent culture conditions, some of the difficulties linked to culture include the cost and the amount of time required for bacterial identification (Seng et al., 2009). These difficulties have recently been overcome by mass spectrometry, which enables quick and effective identifications in routine bacteriology (Seng et al., 2009, 2010) and allow the researcher to quickly check the major population and to concentrate interest on the minority population. We have recently reported a breakthrough in this field of research with the microbial culturomics concept (Lagier et al., 2012a). We applied 212 different culture conditions in two African stools and a French obese stool samples, including enrichment techniques, *Escherichia coli* phage cleaning, and innovative conditions (using rumen fluid, sterile human stools). We analyzed 32,500 colonies by MALDI-TOF, allowed us to culture 340 different bacterial species among seven phyla and 117 genera. This included 174 species never described in the human gut. Moreover 31 new species were found, including five new genera, as well two species from rare phyla (*Deinococcus-Thermus* and *Synergistetes*). Genome sequencing and description of each new species is in progress (Kokcha et al., 2012; Lagier et al., 2012b,c; Mishra et al., 2012a,b,c,d). By comparison, pyrosequencing of 16S rDNA amplicons from the three stools noted a dramatic discrepancy with culturomics as only 51 species identified by 16S rDNA amplification and sequencing were also found among the 340 cultured species highlighting the renewed interest for the culture in the gut microbiota study. Culturomics allowed us break several “records” with the largest number of bacteria cultured from a single stool (219 species), the first bacteria from *Deinococcus-Thermus* phylum isolated from human, the largest human virus and the largest bacteria from human (Lagier et al., 2012a).

### Comparison of the techniques

**Figure 4:**
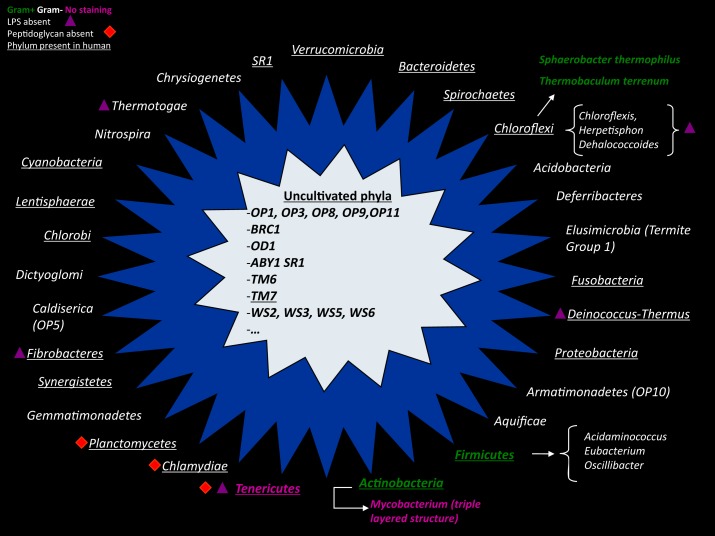
**A non-exhaustive representation of different bacterial phyla found in culture (outer star in blue) or phyla with no representative in culture (inner star in gray)**. Gram-positive bacteria are colored in green, and Gram-negative bacteria are colored in white. Bacteria with an atypical cell wall (triple-layered structure of *Mycobacterium*) or without a cell wall (*Tenericutes*) have abnormal Gram staining and are shown in pink. The purple triangle represents the absence of lipopolysaccharide in the outer membrane of Gram-negative bacteria. The red square symbolizes phyla that do not have a peptidoglycan structure.

There are currently no rational explanations for the typical observed proportions of gram-positive/negative bacteria, which are highly divergent microscopically (Turnbaugh et al., 2007) with culture, (Gossling and Slack, 1974) and the proportions obtained by sequence detection (Eckburg et al., 2005; Figures 2 and 4). In 2002, Hayashi compared the digestive microbiota of three individuals by cloning/sequencing and anaerobic culture using the “plate-in-bottle” method. These researchers isolated between 48 and 65 phylotypes in the cloning of individuals and 48 species, of which three individuals were potentially three new species (Hayashi et al., 2002b). In light of the phylogenetic tree described in this publication, these authors found significant discrepancies between these two techniques, which were somewhat surprising given the low number of species and phylotypes identified. Several species in culture had no equivalent in cloning. A previous study compared these same techniques, but the number of species and phylotypes was even lower (Wilson and Blitchington, 1996). In this study, of 48 species, 25 were detected only by cloning, nine were common to both techniques, and 14 were identified only by culture. In addition, in our microbial culturomics study, by comparison with the 340 bacteria cultured, pyrosequencing of 16S rDNA amplicons from the three stools identified 698 phylotypes including 282 phylotypes of known bacterial species and 416 phylotypes of uncultured bacteria. We noted a dramatic discrepancy with culturomics as only 51 species identified by 16S rDNA amplification and sequencing were also found among the 340 cultured species. Consequently, microbial culturomics increased by 30% the microbial repertoire of the human gut studied by pyrosequencing (Lagier et al., 2012a).

### Gaps in knowledge

**Figure 5:**
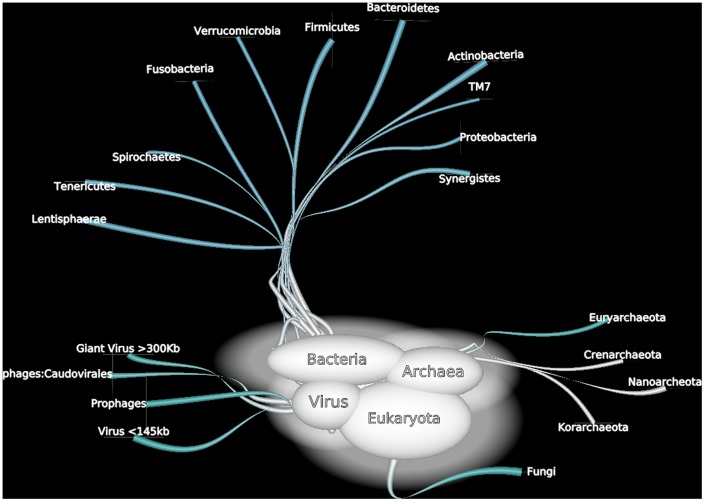
**A non exhaustive overview of human gut microorganisms among bacterial, Archaea, viral, and *Eukaryota* domains**.

In addition to the bias previously described, some components of human gut microbiota have been partially neglected by the current tools (Figure 5).

### Eukaryotes

Eukaryotes are an important part of the human gut microbiome and play different beneficial or harmful roles. Some species may be commensal or mutualistic, whereas others may be opportunistic or parasitic (Parfrey et al., 2011). The eukaryotic component of the human gut microbiome remains unexplored because these organisms are of limited interest (Marchesi, 2010). Culture-dependent techniques and microscopy-based approaches have been mainly used to explore eukaryotes in the human gut, and identification has frequently been based on morphological and physiological techniques with numerous biases. Moreover, this approach detects only a small fraction of microorganisms, including *Candida* and *Saccharomyces* spp., but the growth requirements for many eukaryotic species remain unknown.

Using culture-independent methods, Scupham et al. (2006) have identified a large number of fungi, including *Ascomycota*, *Basidiomycota*, *Chytridiomycota*, and *Zygomycota* phyla, in studies of mouse feces. Furthermore, Scanlan and Marchesi (2008), studying the human distal gut, have shown that the diversity and abundance of eukaryotes is low relative to those of bacteria. Only members of the genera *Gloeotinia*/*Paecilomyces* and *Galactomyces* have been identified as the most abundant. Nevertheless, we have shown that due to a large variety of primers used, the human gut contains a broader eukaryotic diversity than predicted (Hamad et al., 2012). In parallel, applying traditional and modern laboratory techniques (using intergenic spacers for 18S rDNA), the repertoire of intensive care unit pneumonial microbiota has been considerably extended, notably regarding fungal microbiota and plants (Bousbia et al., 2012).

#### Giant viruses

Giant viruses growing in amoebae have previously been isolated in the environment, e.g., in the water of cooling towers, in rivers and lakes, in seawater, in decorative fountains, and in soil (Pagnier et al., 2008). Mimivirus DNA has been obtained from the bronchoalveolar lavage of patients (Raoult et al., 2007; Lysholm et al., 2012), and a laboratory infection was documented by serology (Raoult et al., 2006). In addition, Lysholm et al. (2012), in a viral microbiome metagenomic study performed in 210 children and adults with lower respiratory infections, recently identified Mimivirus. Because the authors used two pools and filtered with 0.22 and 0.45 μm pore-size disk filters, they were able to isolate a giant virus that is frequently missed by large-scale virome metagenomics studies that use only 0.22 μm filters, making giant virus detection unlikely (Willner et al., 2009; Reyes et al., 2010).

In our laboratory, in an effort to obtain fastidious bacteria from an African stool sample by amoeba (*Acanthamoeba polyphaga*) co-culture, we obtained a new giant virus strain named Senegal virus (Lagier et al., 2012a), which we sequenced (Genbank JF909596–JF909602). These findings indicate that giant viruses may be a part of the gut microbiota and that virome metagenomic studies should use different filter sizes. Because the potential pathology of the giant viruses is currently unknown, it is unreasonable to neglect them (Boyer et al., 2009).

#### Archaea

Nottingham and Hungate (1968) isolated a previously unidentified methanogenic Archaea from human feces using a non-selective medium and a stringent anaerobic atmosphere composed of 80% H_2_ and 20% CO_2_. Miller et al. (1982) isolated *Methanobrevibacter smithii* from human stool specimens from four healthy adults using anaerobic cultures enriched with the same H_2_–CO_2_ anaerobic atmosphere pressurized to two bars. Illustrating the technical limitations of the fastidious Archaea culture, in our laboratory, we have recently achieved the isolation of the fourth methanogenic Archaea species in humans and the first cultured representative of a new order of Archaea (*Methanomassiliicoccus* *luminyensis*) after a 16-month tentative culturing procedure. We obtained this strain after subtle modifications in the composition of the culture medium (enzyme co-factors) and adaptation of the atmospheric pressure (the culture medium is patented; Dridi et al., 2012a). In addition, the genome sequencing of this new species represents the largest genome of a methanogenic euryarchaeota isolated from humans (Gorlas et al., 2012).

In addition, recent molecular studies indicated that human Archaea constitute an expanding world (Dridi et al., 2011). Using 16S rDNA sequencing, many studies confirmed the presence of *M. smithii* and *M. stadtmanae* in the human gut, with variable and low prevalence (Dridi et al., 2009). Nevertheless, in our study, our new Archaea was detected in stools in 4% of individuals, and its prevalence increases with age, although its role in human health is unknown (Dridi et al., 2012b). Regarding the influence of Archaea on human health, a recent meta-analysis compared the number of sequences of *Methanobrevibacter* spp. in stools. Obese individuals had fewer *Methanobrevibacter* genera by quantitative polymerase chain reaction (qPCR) than non-obese subjects (Angelakis et al., 2012a). Previous studies had reported discordant results concerning the levels of detection of *M. smithii* in the obese gut (Zhang et al., 2009; Schwiertz et al., 2010; Million et al., 2011). In addition, the detection of Archaea in the vaginal flora of pregnant women allowed us to hypothesize a possible mother-to-child transmission (Dridi et al., 2011).

#### Planctomycetes

The phylum Planctomycetes, phylogenetically closely related to Verrucomicrobia and Chlamydiae, is composed of environmental microorganisms characterized by a peptidoglycan-free cell wall and cell compartmentalization (Fuerst and Sagulenko, 2011). The culture is fastidious and requires the addition of appropriate antibiotics (peptidoglycan synthesis inhibitors) and amphotericin B to prevent contamination of the culture medium. Undetected by conventional 16S rRNA PCR or standard culture techniques, this phylum has been reported in black-and-white colobus monkey stools (Yildirim et al., 2010) and, in one instance, in the human gut microbiota, using metagenomics (De Filippo et al., 2010). In our laboratory, preliminary results (unpublished data) confirmed the presence of specific Planctomycetes DNA in human stools. Several species of Planctomycetes and, more generally, of species including the superphyla Verrucomicrobia, Planctomycetes, and Chlamydiae, are undergoing genome sequencing. It is expected that this sequencing will increase our knowledge of this specific branch of the tree of bacterial life (Wagner and Horn, 2006).

#### The variability depending of the gut samples

“The gut microbiota is non-homogenous with a progressive increase of bacterial concentration from the stomach (approximately 10^3^ bacteria per gram to the colon (approximately 10^11^ bacteria per gram; O’Hara and Shanahan, 2006). Nevertheless, most of studies explored stools samples reflecting mainly the colonic composition. However, differences in compositions have been reported between small intestine biopsies (most of Streptococcaceae belonging to Firmicutes phylum and Actinomycinaeae and Corynebacteriaceae belonging to Actinobacteria phylum) whereas colonic biopsies were enriched by Bacteroidetes phylum and Lachnospiraceae among the Firmicutes phylum (Frank et al., 2007). Intestinal analysis of tiered samples will allow to exhaustively describe the gut composition.”

## Composition

**Figure 6:**
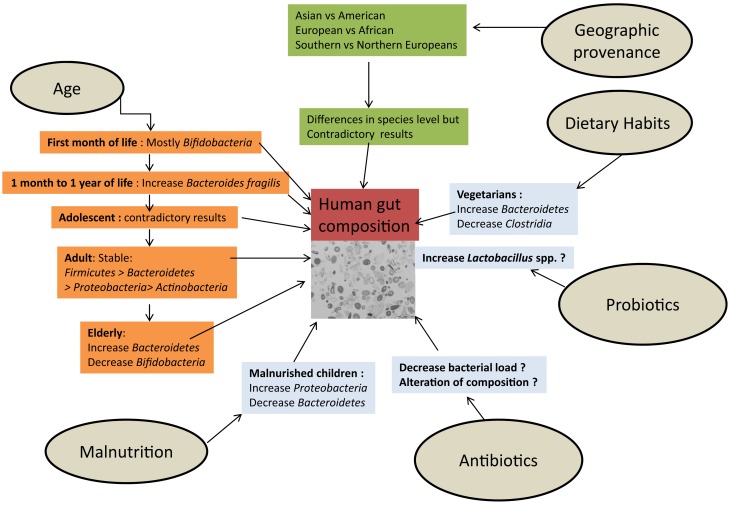
**The influence of external factors determining the composition of the human gut microbiota**.

The composition of the human gut ecosystem is influenced by multiple and diverse factors, some physiological (age, origin, environment) and others linked to external factors, such as dietary habits, antibiotics, and probiotics (Figure 6).

### Age

In a pioneering study using microarrays to detect small rRNAs, Palmer followed a cohort of newborns, including a pair of twins, during the first year of life. It was shown that despite considerable temporal variations and environmental influences, the composition of the intestinal ecosystem tended to be characteristic of adulthood at the end of this period (Palmer et al., 2007). The proportion of *Bacteroides fragilis* increased from 1 month to 1 year (Vael et al., 2011). In a 2.5-year case study, Koenig analyzed >300,000 16S rRNAs from 60 fecal samples from healthy children and showed that infant gut variation is associated with life events. The phylogenetic diversity of the microbiome increased gradually over time with progressive temporal changes but, inversely, the major phyla, genera, and species composition showed rough shifts in abundance corresponding to modifications in diet or health (Koenig et al., 2011).

Nevertheless, using microbiota array to analyze gut microbiota composition in adolescent subjects, Agans et al. (2011) found a statistically significantly higher abundance of *Bifidobacterium* and *Clostridium* genera, contrary to current knowledge, suggesting that the gut microbiome of adolescents is different from that of adults. At the other extreme of life, using pyrosequencing of 16S rRNA gene V4 region amplicons, the gut microbiota composition of elderly subjects was distinct from that of younger adults, with a greater temporal stability over a limited time, particularly in the proportion of *Bacteroides* spp. (Claesson et al., 2011).

### Geographical provenance and environment

Discordant results have also been published regarding a geographic signature of the gut microbiota depending on the technique used. To investigate the hypothetical association between gut composition and cancer, early culture-dependent studies compared populations at high-risk (western countries) and at low risk (Japan, Uganda, India) and reported different compositions of microbiota (Hill et al., 1971; Drasar et al., 1973; Finegold et al., 1974). The high-risk population had a microbiota composed primarily of *Bacteroidetes*, and there were specific differences, including patterns of food consumption, between western countries and Asian or African populations, although multiculturalism and population exchanges have reduced these differences. Only a few large-scale molecular studies have used stool samples collected from Asia or Africa (De Filippo et al., 2010; Lee et al., 2011), where approximately 75% of the population of the world lives; nevertheless, the findings have suggested a possible signature of biogeography (Lee et al., 2011). Indeed, most of the large-scale metagenomic or pyrosequencing studies used stools collected from American or European individuals (Ley et al., 2006b; Turnbaugh et al., 2006; Claesson et al., 2011).

Lay, characterizing 91 European gut microbiota using FISH combined with flow cytometry, did not observe a significant grouping with regard to country of origin (France, Netherlands, Denmark, UK, and Germany; Lay et al., 2005). With the same technology, Mueller et al. (2006) found differences in *Bifidobacteria* species between European individuals. Grzeskowiak et al. (2012), using flow cytometry-FISH and qPCR, have shown that several species (*Bifidobacterium adolescentis*, *Staphylococcus aureus*, and *Clostridium perfringens*) were absent in Malawian children but present in 6-month-old Finnish infants. Fallani comparing infants living in northern or southern European countries by 16S rDNA pyrosequencing, have found that geographical provenance is important, with a higher proportion of *Bifidobacteria* in northern infants and more *Bacteroidetes* and *Lactobacilli* in southern European countries.

Finally, Arumugam studied 22 fecal metagenomes of individuals from four different countries and identified three different enterotype clusters, which were independent of geographic provenance. The three different enterotypes were, respectively, richer in *Bacteroides*, in *Prevotella*, and in *Ruminococcus* for the Enterotype 3. Arumugam suggested that each enterotype used a different route to generate energy (Arumugam et al., 2011).

### Dietary habits

Dietary habits are thought to be a major factor contributing to the diversity of the human digestive microbiota (Backhed et al., 2005). Part of the geographic diversity of the gut microbiota seems to be explained by differences in diet. For example, African children from a rural area in Burkina Faso showed a specific abundance of *Prevotella* and *Xylanibacter*, known to contain a set of bacterial genes for cellulose and xylan hydrolysis, completely lacking in European children (De Filippo et al., 2010). The authors hypothesized that the abundance of these genera could be a consequence of the high intake of fiber, similar to the diet of early human settlements at the time of the birth of agriculture, maximizing the extraction of metabolic energy from the polysaccharides of ingested plants (De Filippo et al., 2010). A vegetarian diet affects the intestinal microbiota, specifically by decreasing the amount and modifying the diversity of *Clostridium* cluster IV (Liszt et al., 2009). Based their studies on RFLP analysis, Hayashi et al. (2002a) Hayashi found that the digestive microbiota of vegetarians harbored Clostridium rRNA clusters XIVa and XVIII. Recently, Walker et al. (2011) tested overweight people successively with a control diet, a diet high in resistant starch (RS) or non-starch polysaccharides (NSP) and a reduced carbohydrate weight loss (WL) diet for 10 weeks by two different methods: large-scale sequencing and quantitative PCR. No significant effect was observed at the phylum level, but at finer taxonomic level, *Eubacterium* *rectale* and *Ruminococcus bromii* showed significant and dramatic (fourfold) increased proportions in the RS diet, whereas the proportion of *Collinsella aerofaciens*-related sequences was decreased significantly on the WL diet (Walker et al., 2011). In this study, reproducible changes were found only at the phylotype level, whereas no differences were significant at a broader taxonomic level (the phylum or family Ruminococcaceae level), and the analysis suggested that the amplified 16S rRNA sequence clustered more strongly by individuals than by diet. These changes are entirely in agreement with studies using RNA-based stable isotope probing, which showed that *R. bromii* was the first starch degrader in the human gut (Kovatcheva-Datchary et al., 2009). Wu analyzed stool samples from 98 individuals and found that enterotypes were strongly associated with long-term diets, especially for animal fat and protein (*Bacteroides*) vs. carbohydrate (*Prevotella*). Changes in gut microbiota related to short-term diet modifications occurred rapidly, were detectable within 3–4 days, and were rapidly reversed (Walker et al., 2011; Wu et al., 2011). Conversely, Wu et al. (2011) suggested that long-term dietary interventions might allow the pervasive modulation of an individual’s enterotype to improve health. In animal models of obesity induced by diet (DIO), Turnbaugh et al. (2008) showed that a high-fat diet could significantly alter the intestinal flora of experimental models with a bloom in a single uncultured clade within the *Mollicutes* class of the *Firmicutes*. Hildebrandt et al. (2009) suggested that a high-fat diet altered the intestinal flora regardless of weight change. These authors observed a bloom of *Clostridia* and *Proteobacteria* associated with the high-fat diet. The major group of *Proteobacteria* that increased in abundance was the Delta-*Proteobacteria* phylum, order *Desulfovibrio*. Finally, Monira et al. (2011) have recently published a study comparing the gut flora of malnourished children with that of well-nourished children in Bangladesh and found a decrease in *Bacteroidetes* and an increase in *Proteobacteria* phyla, including *E. coli* and *Klebsiella* spp.

### Obesity and gnotobiotic mice

Beginning in 2005, obesity has been associated with a specific profile of bacterial gut microbiota, including a decrease in the *Bacteroidetes/Firmicutes* ratio (Ley et al., 2005, 2006b; Turnbaugh et al., 2006, 2009) and decreased bacterial diversity (Turnbaugh et al., 2009). Since these pioneering studies, significant associations have been found between obesity and an increase in some bacterial groups, including *Lactobacillus*, *S. aureus, E. coli*, and *Faecalibacterium prausnitzii* (Collado et al., 2008; Kalliomaki et al., 2008; Armougom et al., 2009; Santacruz et al., 2009; Balamurugan et al., 2010). In a recent review, we found no reproducible and significant alteration linking obesity and gut microbiota at the phylum level (Angelakis et al., 2012a). Conversely, meta-analysis at the genus-level found decreased levels of bifidobacteria (Collado et al., 2008; Kalliomaki et al., 2008; Santacruz et al., 2009; Balamurugan et al., 2010; Schwiertz et al., 2010) and *Methanobrevibacter* spp. (Armougom et al., 2009; Schwiertz et al., 2010; Million et al., 2011), the leading known representative of Archaea in the human gut, in overweight/obese people. To date, controversial studies show that the connection between the microbiome and excess weight is complex (Pennisi, 2011). We found a difference at the species level, with *L. reuteri* enriched in obese gut microbiota, whereas *L. plantarum* was increased in lean individuals (Million et al., 2011). At the gene level, obesity has been associated with an altered representation of bacterial genes and metabolic pathways. Turnbaugh et al. (2009) showed that diversity of organismal assemblages yields a core microbiome at a functional level and that deviations from this core are associated with different physiological states (obese compared with lean), with obese gut microbiota having an increased capacity for energy harvest.

As a theoretical basis for the causal link between alterations in the gut microbiota and obesity, several mechanisms have been suggested. First, the gut microbiota may interact with weight regulation by hydrolyzing indigestible polysaccharides to easily absorbable monosaccharides and by activating lipoprotein lipase. Consequently, glucose is rapidly absorbed, producing substantial elevations in serum glucose and insulin, both factors that trigger lipogenesis. In addition, fatty acids are stored excessively, with *de novo* synthesis of triglycerides derived from the liver. Together, these phenomena cause weight gain (Backhed et al., 2007). Using *Fasting-Induced Adipocyte Factor* (Fiaf) knockout mice, Backhed et al. (2007) showed that gut microbiota suppressed intestinal Fiaf, consequently increasing the storage of calories.

Second, the composition of gut microbiota has been shown to selectively suppress angiopoietin-like protein 4/fasting-induced adipose factor in the intestinal epithelium. This molecule is a circulating lipoprotein lipase inhibitor and a regulator of peripheral lipid and glucose metabolism (Backhed et al., 2004). Backhed et al. (2004) showed that when the microbiota of normal mice were transplanted into germ-free mice, after 2 weeks, body fat increased by 60% without increased food consumption, modifications of energy expenditure, or relative insulin resistance, and there was a 2.3-fold higher production of triglycerides in the liver, suggesting that the gut operates in host energy homeostasis and adiposity.

Third, it has been suggested that bacterial isolates of gut microbiota may have pro- or anti-inflammatory properties, impacting weight. Obesity has been associated with a low-grade systemic inflammation corresponding to higher plasma endotoxin lipopolysaccharide (LPS) concentrations, defined as metabolic endotoxemia (Bastard et al., 2006; Hotamisligil, 2006; Sbarbati et al., 2006; Fogarty et al., 2008). Cani et al. (2008) showed that antibiotics can lower LPS levels in mice fed a high-fat diet and in *ob/ob* mice and, consequently, can reduce glucose intolerance, body weight gain, and fat mass. Conversely, some *Bifidobacterium* and *Lactobacillus* species have been reported to deconjugate bile acids, which may decrease fat absorption (Shimada et al., 1969).

### Antibiotics and probiotics

#### Antibiotics and total bacterial count

According to the literature, oral or intravenous antibiotics tend to decrease the bacterial load in the digestive tracts of infants (Palmer et al., 2007) and elderly patients (Bartosch et al., 2004). However, other studies reported that only the microbiota composition is altered, and the total biomass is not modified by antibiotics (Sekirov et al., 2008). In contrast to amoxicillin and metronidazole or cefoperazone, Robinson noted that the alterations in community structure associated with vancomycin specifically occurred without a significant decrease in the overall bacterial biomass (Robinson and Young, 2010).

#### Structural disruption

Antibiotic administration has a reproducible effect on the community structure of the indigenous gastrointestinal microbiota in mice (Robinson and Young, 2010). A very recent study found that the administration of a commercial growth-promoting antibiotic combination (ASP250: chlortetracycline-sulfamethazine and penicillin) entailed a reproducible bloom in proteobacteria (1–11%) in swine gut microbiota (Looft et al., 2012). This shift was driven by an increase in *E. coli* populations. In humans, analysis of the fecal microbial populations of infants after antibiotic therapy showed a major alteration as measured by SSU rDNA microarray analysis (Palmer et al., 2007) or culture-based methods (Savino et al., 2011). In adults, the same dramatic shift has been reported, depending on the antibiotic. Clindamycin (Donskey et al., 2003; Jernberg et al., 2005a) has the strongest effect compared to oral cephalosporin, which is responsible for minor or no changes (Swedish Study Group, 1991a,b). Of note, the extremely moderate effect of cephalosporin on gut microbiota (Donskey et al., 2003) has been linked with the low activity of this molecule on intestinal anaerobes. Moreover, the fecal elimination of carbapenems is very limited, explaining why changes in the intestinal microflora are only moderate, whereas these agents have the broadest spectra of the beta-lactam antibacterial agents (Sullivan et al., 2001). The characterization of gut microbiota alteration by metagenomic analysis of the v3–v6 region has been studied in three patients on ciprofloxacin (Dethlefsen et al., 2008). Ciprofloxacin decreased to one-third the abundance of taxa [number of ref Operational taxonomic units (OTU)], their diversity and distribution. However, comparing gut microbiota alterations by DGGE analysis, the rate of similarity with the pre-treatment profile was 73% with ciprofloxacin but only 11–18% with clindamycin (Donskey et al., 2003). In addition, ciprofloxacin has been reported to have little or no impact on anaerobic intestinal microbiota (Nord, 1995; Edlund and Nord, 1999b). Glycopeptides, used widely in agriculture as growth promoters, are associated with natural resistance of most of the lactobacilli and have no effect on gram-negative bacteria, including *Enterobacteria* (Barna and Williams, 1984). Analyzing vancomycin-associated gut microbiota alterations in mice by cloning sequencing, Robinson found that vancomycin increased members of the *Proteobacteria* and *Tenericutes* phyla and the Lactobacillaceae family, whereas members of the Lachnospiraceae family decreased (Robinson and Young, 2010). Using a continuous-culture colonic model system, Maccaferri et al. (2010) demonstrated that rifaximin, reported to induce clinical remission of active Crohn’s disease while not altering the overall structure of the human colonic microbiota, increased *Bifidobacterium*, *Atopobium*, and *F. prausnitzii* and led to a variation of metabolic profiles associated with potential beneficial effects on the host. The effects of tetracycline on gut microbiota in humans are of particular interest because this antibiotic is commonly used in poultry production as a growth promoter, suggesting dramatic changes in intestinal microbial populations. One notable effect of tetracycline is a decrease in bifidobacteria (Nord et al., 2006; Saarela et al., 2007). Overall, specific gut microbiota changes are associated with specific antibiotics (Table 1).

**Table 1:** **Modifications of gut flora linked to antibiotics**.

Antibiotic	Method	References
**PENICILLINS**		
**Ampicillin**		
Decrease in enterococci	Cultivation	Black et al. (1991)
Decrease in streptococci	Cultivation	Black et al. (1991)
Decrease in *E. coli* strains	Cultivation	Black et al. (1991)
Slight decrease in anaerobic Gram-positive bacteria	Cultivation	Black et al. (1991)
**Amoxicillin**		
Increase in aerobic Gram-negative rods, such as enterobacteria, other than *E. coli* (*Klebsiella*, *Enterobacter*)	Cultivation	Brismar et al. (1993), Floor et al. (1994), Stark et al. (1996)
Increase in anaerobic Gram-positive rods	Cultivation	Swedish Study Group (1991b)
Increase in *Bacteroides*	Cultivation	Swedish Study Group (1991b)
Decrease in streptococci and Staphylococci	Cultivation	Brismar et al. (1993)
Decrease in anaerobic Gram-positive cocci, such as eubacteria	Cultivation	Brismar et al. (1993), Stark et al. (1996)
**Amoxicillin/clavulanic acid**		
Increase in enterococci and *E. coli*	Cultivation	Lode et al. (2001)
Decrease in lactobacilli, clostridia, bifidobacteria	Cultivation	Lode et al. (2001)
Disappearance of *Clostridium* cluster XIVa (cloning/sequencing)	Cloning/sequencing	Young and Schmidt (2004)
Decrease in *Faecalibacterium* spp.	Cloning/sequencing	Young and Schmidt (2004)
Piperacillin/tazobactam*		
Decrease in enterobacteria	Cultivation	Nord et al. (1993)
Decrease in bifidobacteria, eubacteria, lactobacilli	Cultivation	Nord et al. (1993)
Decrease in anaerobic Gram-positive cocci like clostridia	Cultivation	Nord et al. (1993)
**CEPHALOSPORINS**		
**Cefepime**		
Decrease in *E. coli* and bifidobacteria	Cultivation	Bacher et al. (1992)
Increase in clostridia and *Bacteroides*	Cultivation	Bacher et al. (1992)
**Ceftriaxone**		
Decrease in the total numbers of anaerobes	Cultivation	Welling et al. (1991)
Dramatic decrease in clostridia, lactobacilli, bifidobacteria	Cultivation	Vogel et al. (2001)
Dramatic decrease in Gram-negative rods (enterobacteria)	Cultivation	Cavallaro et al. (1992), Vogel et al. (2001), Welling et al. (1991)
Increase in enterococci	Cultivation	Vogel et al. (2001), Welling et al. (1991)
**Carbapenems**		
**Meropenem**		
Decrease in enterobacteria and streptococci	Cultivation	Bergan et al. (1991)
Increase in enterococci	Cultivation	Bergan et al. (1991)
Decrease in clostridia, Gram-negative cocci, and bacteroides	Cultivation	Bergan et al. (1991)
**FLUOROQUINOLONES**		
**Ciprofloxacin**		
Dramatic decrease in enterobacteria	Cultivation	Bergan et al. (1986), Borzio et al. (1997), Brismar et al. (1990), Brumfitt et al. (1984), Enzensberger et al. (1985), Esposito et al. (1987), Holt et al. (1986), Krueger et al. (1997), Ljungberg et al. (1990), Rozenberg-Arska et al. (1985), Van Saene et al. (1986), Wistrom et al. (1992)
Decrease in aerobic Gram-positive cocci	Cultivation	Bergan et al. (1986), Brismar et al. (1990), Brumfitt et al. (1984), Ljungberg et al. (1990), Van Saene et al. (1986)
Decrease in streptococci	Cultivation	Brismar et al. (1990), Brumfitt et al. (1984), Ljungberg et al. (1990)
Decrease in enterococci	Cultivation	Bergan et al. (1986), Brismar et al. (1990), Ljungberg et al. (1990), Van Saene et al. (1986)
Increase in enterococci	Cultivation	Borzio et al. (1997)
Decrease in anaerobic bacteria	Cultivation	Bergan et al. (1986), Brismar et al. (1990), Rozenberg-Arska et al. (1985)
Suppression of *Bacteroides putredinis*, *Ruminococcus torques*	DGGE	Donskey et al. (2003)
**Norfloxacin**		
Dramatic decrease in enterobacteria	Cultivation	de Vries-Hospers et al. (1985), Edlund et al. (1987), Leigh et al. (1985), Pecquet et al. (1986)
Decrease in aerobic Gram-positive cocci	Cultivation	de Vries-Hospers et al. (1985), Pecquet et al. (1986)
Decrease in streptococci	Cultivation	Pecquet et al. (1986)
Decrease in enterococci	Cultivation	de Vries-Hospers et al. (1985)
**Ofloxacin**		
Dramatic decrease in enterobacteria	Cultivation	Edlund et al. (1988), Edlund et al. (1997b), Pecquet et al. (1987)
Decrease in aerobic Gram-positive cocci	Cultivation	Edlund et al. (1988), Edlund et al. (1997b), Pecquet et al. (1987)
Decrease in enterococci	Cultivation	Edlund et al. (1988), Pecquet et al. (1987)
Decrease in lactobacilli, bifidobacteria, eubacteria	Cultivation	Edlund et al. (1988)
Decrease in anaerobic bacteria	Cultivation	Edlund et al. (1988)
Decrease in *Veillonella* and *Bacteroides* spp.	Cultivation	
**Levofloxacin, Gatifloxacin, Trovafloxacin, Moxifloxacin**		
Dramatic decrease in enterobacteria	Cultivation	Edlund et al. (1997b), Edlund and Nord (1999a), van Nispen et al. (1998)
Strong decrease in aerobic Gram-positive cocci	Cultivation	Edlund et al. (1997b), Edlund and Nord (1999a), van Nispen et al. (1998)
Levofloxacin, gatifloxacin: decrease in clostridia	Cultivation	Edlund et al. (1997b), Edlund and Nord (1999a)
Gatifloxacin: decrease in fusobacteria	Cultivation	Edlund and Nord (1999a)
**GLYCOPEPTIDS**		
**Oral vancomycin**		
Decrease in enterococci	Cultivation	Edlund et al. (1997a), Lund et al. (2000)
Decrease in staphylococci	Cultivation	Van der Auwera et al. (1996)
Overgrowth of lactobacilli (and pediococci)	Cultivation	Edlund et al. (1997a), Lund et al. (2000), Van der Auwera et al. (1996)
Strong suppression or elimination of bacteroides	Cultivation	Edlund et al. (1997a), Lund et al. (2000)
Decrease in clostridia and bifidobacteria	Cultivation	Lund et al. (2000)
**Oral teicoplanin**		
Increase in Gram-negative aerobic rods and total numbers of aerobes	Cultivation	Van der Auwera et al. (1996)
Increase in lactobacilli and pediococci	Cultivation	Van der Auwera et al. (1996)
**LINEZOLID**		
Reduction of enterococci	Cultivation	Lode et al. (2001)
Reduction of bifidobacteria, lactobacilli, clostridia, and bacteroides	Cultivation	Lode et al. (2001)
Increase in *Klebsiella*	Cultivation	Lode et al. (2001)
**TETRACYCLINES**		
**Doxycycline**		
Decrease in bifidobacteria	Cultivation	Saarela et al. (2007)
**Tigecycline**		
Decrease in enterococci	Cultivation	Nord et al. (2006)
Decrease in *E. coli*	Cultivation	Nord et al. (2006)
Increase of other enterobacteria (*Klebsiella* and *Enterobacter* spp.)	Cultivation	Nord et al. (2006)
Marked reduction of lactobacilli and bifidobacteria	Cultivation	Nord et al. (2006)
Increase in yeasts	Cultivation	Nord et al. (2006)
**MACROLIDES, LINCOSAMIDES, SYNERGISTINS**		
**Erythromycin**		
Dramatic decrease in streptococci and enterobacteria	Cultivation	Brismar et al. (1991)
Decrease in clostridia, lactobacilli, bifidobacteria, and bacteroides	Cultivation	Brismar et al. (1991)
**Clarithromycin**		
Reduction of enterobacteria, *E. coli*, and streptococci	Cultivation	Brismar et al. (1991), Edlund et al. (2000b)
Dramatic decrease in clostridia, and bacteroides	Cultivation	Brismar et al. (1991)
Reduction of lactobacilli and bifidobacteria	Cultivation	Brismar et al. (1991), Edlund et al. (2000b)
**Telithromycin**		
Decrease in *E. coli* but overgrowth of non-*E. coli* enterobacteria	Cultivation	Edlund et al. (2000a)
Reduction of lactobacilli and bifidobacteria	Cultivation	Edlund et al. (2000a)
**Clindamycin**		
Increase in enterobacteria	T-RFLP	Jernberg et al. (2005b)
Decrease in total anaerobic bacteria	Cultivation	Nord et al. (1997)
Decrease in lactobacilli and *Bacteroides*	Cultivation	Nord et al. (1997), Sullivan et al. (2003)
Decrease in clostridia	Cultivation	Nord et al. (1997)
Disappearance of bifidobacteria	Cultivation	Jernberg et al. (2005b), Nord et al. (1997)
Dramatic decrease in *Bifidobacterium*, *Clostridium* (particularly *C. coccoides* subgroup as *Eubacterium*) and *Bacteroides*	T-RFLP	Jernberg et al. (2005b)
Suppression of *B. vulgatus*, *B. acidofasciens*, *F. prausnitzii*, *C. indolis*, and *C. leptum* cluster	DGGE	Donskey et al. (2003)
No change in *B. thetaiotaomicron* and *B. uniformis*	DGGE	Donskey et al. (2003)
**Streptogramins: Quinupristin/dalfopristin**		
Decrease in anaerobic Gram-negative bacteria	Cultivation	Scanvic-Hameg et al. (2002)
Increase in enterococci and enterobacteria	Cultivation	Scanvic-Hameg et al. (2002)
**OTHERS**		
**Cotrimoxazole**		
Suppression of Enterobacteriaceae	Cultivation	Mavromanolakis et al. (1997)
**Metronidazole**		
No significant change but not enough data available	Cultivation	Sullivan et al. (2001)
**Nitrofurantoin**		
No impact on intestinal microflora	Cultivation	Mavromanolakis et al. (1997)

The effects of three growth-promoting antibiotics (avilamycin, zinc bacitracin, and flavomycin) on broiler gut microbial community colonization and bird performance were investigated (Torok et al., 2011). OTU linked to changes in gut microbiota in birds on antimicrobial-supplemented diets were characterized and identified. Lachnospiraceae, *L. johnsonii*, Ruminococcaceae, and Oxalobacteraceae genera were less prevalent in the guts of chicks fed antimicrobial-supplemented diets. *L. crispatus*, *L. reuteri*, *Subdoligranulum*, and Enterobacteriaceae were more prevalent in the guts of chicks raised on the antimicrobial diet (Torok et al., 2011). These results suggest that antibiotic effects on gut microbiota may be relevant at the species level because different *Lactobacillus* species-related OTUs showed paradoxical changes.

#### The reversibility of structural gut microbiota modification

The recovery of the gut community toward baseline after short-term antibiotic therapy has been reported in animal models (Robinson and Young, 2010), but pervasive disturbance to the community has been observed several weeks after withdrawal of certain antibiotics, including cefoperazone (Robinson and Young, 2010) and quinolones (Dethlefsen et al., 2008). Changing the intestinal microbiota of termites with antibiotics offers a privileged experimental model and has shown that prolonged antibiotic treatment with rifampicin has an irreversible effect not only on microbial diversity but also on longevity, fecundity and the weight (weight gain compared to controls) of two termite species, *Zootermopsis angusticollis* and *Reticulitermes flavipes*.

#### Probiotics

Probiotics were initially used in agriculture to prevent diarrhea in poultry because they reduce intestinal colonization by *Salmonella* spp. and *C. perfringens* (Angelakis and Raoult, 2010), but the use of probiotics such as *Lactobacillus* spp. can led to a rapid weight increase in chickens (Angelakis and Raoult, 2010). *L. acidophilus*, *L. plantarum*, *L. casei*, *L. fermentum*, and *L. reuteri* are the most commonly used *Lactobacillus* species in agriculture (Anadon et al., 2006). The inoculation of *L. ingluviei* in mice is responsible for gut flora alterations associated with an increase in weight gain and liver enlargement (Angelakis et al., 2012b). In parallel, probiotics are increasingly used in human foods, notably in the milk industry (Raoult, 2008). Although the mechanisms are not yet known, many studies suggest that probiotics function through direct or indirect impacts on colonizing microbiota of the gut (Sanders, 2011). Million et al. (2011) recently found that different *Lactobacillus* species may have a paradoxical effect, with higher levels of *L. reuteri* and lower levels of *L. plantarum* and *L. paracasei* in obese gut microbiota. A recent systematic meta-analysis reported that the administration of *L. acidophilus* is responsible of weight gain in human and animals and that the use of *L. fermentum* and *L. ingluviei* resulted of weight gain in animals (Million et al., 2012). Thuny et al. (2010) observed a weight gain in patients treated with vancomycin and hypothesized that the gain was induced by the growth-promoting effect of *Lactobacillus* spp., as these species are resistant to glycopeptides. In contrast, symbiotics (the combination of prebiotics and probiotics) have been proposed for the management of malnutrition, with promising results on mortality (Kerac et al., 2009). After gavage of gnotobiotic mice with a combination of bacteria, including *B. animalis* subsp. *lactis*, *L. delbrueckii* subsp. *bulgaricus*, *Lactococcus lactis* subsp. *cremoris*, and *Streptococcus thermophilus*, only anecdotal changes were noted in microbiota composition, whereas significant changes were observed in the expression of microbiome-encoded enzymes involved in metabolic pathways, notably, carbohydrate metabolism (McNulty et al., 2011). However, these suggestions of a relationship between probiotics and obesity remain controversial (Delzenne and Reid, 2009). In addition, the reports of the anti-diabetic and anti-inflammatory effects of *Lactobacilli* should be considered cautiously because the translation of findings based on animal models to humans is hazardous (Kootte et al., 2012). Finally, all these results should be interpreted with caution in view of the substantial funding of obesity research by the food industry, creating conflicts of interest.

## Reductionist Approach and Biases

In their attempts to reduce ignorance and in contrast to the holistic approach based on the combination of conventional techniques and technology-driven methods, which enable researchers to study and make sense of a complex ecosystem, diverse studies based on experimental models have induced reductionism in the understanding of human microbiota and have generated contradictory results (Raoult, 2010; Fang and Casadevall, 2011).

### Hypothesis-driven research versus holistic-driven research

Because it has been suggested that gut microbiota play a role in health and disease, it has been attractive to find a stable model to help scientists to understand host-gut microbiota mutualism, but this relationship is very complex and involves control diets, genetics, and environmental conditions. The first germ-free model was used by Pasteur in 1885. Since that time, various models have been used to study gut microbiota, including germ-free neonatal pigs (Meurens et al., 2007), zebrafish (Rawls et al., 2004), and gnotobiotic mice, which is the most effective tool (Backhed et al., 2007; Goodman et al., 2011).

Conversely, based on observations and not supported by any preconceived hypothesis, several findings by different research teams have shown a significant reduction in *Bacteroidete*s proportions in obese patients (Armougom et al., 2009; Turnbaugh et al., 2009; Million et al., 2011). Comparing the composition of the gut microbiota between young adult female monozygotic or dizygotic twins who are obese or lean and their mothers, Turnbaugh found that obesity was associated with reduced bacterial diversity and, notably, a reduced proportion of *Bacteroidetes*. Moreover, the genes over-represented within obese individuals exclusively belong to the *Firmicutes* phylum (Turnbaugh et al., 2009). Nevertheless, previous investigations based on genomic data and experimental models have shown that co-colonization of *B*. *thetaiotaomicron* and *M. smithii* in the digestive tract of xenobiotic mice was responsible for significant weight gain, with discordance between sequence analysis results and the initial hypothesis (Xu et al., 2003; Samuel and Gordon, 2006).

### Conflicts of interest

Finally, to exhaustively review the topic of human gut microbiota composition and mutualism with the host, it would be morally objectionable not to address the central influence of funding sources and transparency disclosures (Million and Raoult, 2012).

A transparency declaration of conflict of interest is important for publication in the medical literature. Lundh et al. (2010), based on the articles published in six of the most prestigious medical journals, showed that the publication of studies financed by industries was associated with an increase in the impact factor of the journal. Regarding the economic aspect and the payment of physicians by five manufacturers of hip and knee prostheses, a recent study confirmed that only approximately 80% of direct payments and 50% of indirect payments to physicians have been disclosed (Okike et al., 2009). Some authors even consider that publications in medical journals are a marketing tool for the pharmaceutical industry (Smith, 2005). In the beverage and food industry, Levine studied the financial relationships between industry and authors who have published research on alimentary substitutes. Classifying these publications as neutral, critical, or supportive toward the alimentary substitutes, the authors suggested a significant association between the authors who support the efficiency of the substitute and the authors with financial relationships with the industrial company (Levine et al., 2003).

Finally, Thomas et al. (2008) has recently shown that of 63 randomized trials published regarding nutrition and obesity, 67% were supported by the food industry. Moreover, the results of industry-supported trials were significantly associated with a higher quality of reporting score associated with long-term WL. Moreover, compounding this problem, some scientists do not declare their conflicts of interest. Based on these data, and the considerable financial involvement associated with human gut microbiota research, notably in obesity, we regret that there is not more public funding (Smith, 2005), and that conflict of interest with food industry are not actively required as for pharmaceutical industry.

## Concluding Remarks

Factors affecting the composition of the gut microbiota and the relationship with the hosts are of considerable complexity. Both physiological and external factors are often unstable over time, influencing the gut microbiota. Despite the contribution of recent technologies, the repertoire of this ecosystem remains incomplete. As a striking example, despite the dramatic increase in the number of publications regarding gut microbiota, simplistic anomalies persist, such as the discordance among microscopic observation, pyrosequencing, and culture results. We regret that fewer studies are based on observation and description in opposition to studies performed to confirm a hypothesis. Indeed, it is paradoxical to design experiments and models to confirm a hypothesis because the ecosystem is only partially described. Finally, the central problem of funding sources and transparency declarations lead us to hope that public funding will develop food-industry-independent research to increase confidence in the results.

In the future, we think that culturomics followed by the high-throughput genome sequencing and its applications as the exploration of host-pathogen interactions will allows to capture the relationships in the gut microbiota. In addition, technology advances in pyrosequencing with higher reads fragment analysis, may facilitate the analysis to low taxonomic level (genera, species) reducing consequently the depth bias.

## Conflict of Interest Statement

The authors declare that the research was conducted in the absence of any commercial or financial relationships that could be construed as a potential conflict of interest.
